# Impact of Laparoscopic Sleeve Gastrectomy on Thrombomodulin Concentration and Early Markers of Atherosclerosis

**DOI:** 10.1155/2022/6152571

**Published:** 2022-07-08

**Authors:** Hasan Elkan, Mehmet Memduh Baş, Berna Kaya

**Affiliations:** ^1^Department of General Surgery, University of Harran, Sanliurfa, Turkey; ^2^Department of Cardiology, Private Meydan Hospital, Sanliurfa, Turkey; ^3^Department of Internal Medicine, University of Health Sciences Mehmet Akif Inan Training and Research Hospital, Sanliurfa, Turkey

## Abstract

**Background:**

Thrombomodulin, an integral membrane protein functioning as a cofactor in the anticoagulant pathways, has recently emerged as a marker of endothelial dysfunction. This study aimed to investigate the impact of laparoscopic sleeve gastrectomy (LSG) on thrombomodulin concentration and early markers of atherosclerosis.

**Methods:**

Forty-four subjects undergoing LSG were prospectively examined. The change in thrombomodulin concentration from baseline (preoperative) to the sixth postoperative month following the LSG and the relationship between the change in thrombomodulin concentration and BMI, CIMT, ABI, and blood lipids were examined.

**Results:**

The medical records were available for 44 patients (mean age: 37.2 ± 10.9 years, 65.9% male). LSG led to significant reductions in total body weight and body mass index (BMI) at postoperative six months (37.0 ± 5.6 kg/m^2^ vs. 47.1 ± 5.8 kg/m^2^, *p* < 0.001). Markers of early atherosclerotic events, including carotid intima-media thickness (CIMT) and ABI, improved. The change in thrombomodulin concentration (Δ TMD) was significantly correlated with the change in Δ BMI (*r* = 0.500, *p*=0.011), Δ LDL (*r* = 0.389, *p*=0.032), Δ systolic blood pressure (*r* = 0.384, *p*=0.012), and Δ CIMT (*r* = 0.327, *p*=0.012) and was negatively correlated with Δ HDL (*r* = −0.344, *p*=0.020) and Δ ABI (*r* = −0.357, *p*=0.020).

**Conclusion:**

LSG leads to significant improvements in blood lipids, systolic and diastolic blood pressure, and in surrogate markers of atherosclerotic burden and endothelial function, including thrombomodulin, ABI, and CIMT, at postoperative six months. LSG might prevent or reduce atherogenesis in the early stages by stopping endothelial dysfunction.

## 1. Introduction

Coronary artery disease (CAD) is one of the most frequent causes of morbidity and mortality worldwide. Age, smoking, diabetes, hypertension, and hypercholesterolemia are well-documented risk factors for the development of CAD. Obesity has recently gained emphasis as a common risk factor for CAD, particularly in developed countries. The prevalence of being overweight and obese in the USA is 69% and 35%, respectively [1]. Obesity has been reported to result in CAD either as an independent risk factor or indirectly by leading to hypertension, diabetes, or hyperlipidemia [2]. Recent evidence indicates that adipose tissue may induce atherosclerotic vascular disease by producing a wide range of adipokines directly involved in the atherosclerotic process [3]. It has been reported that the risk of CAD increases by 12% with every 10 kg increment in body weight [4]. Individuals with a higher body mass index tend to have more frequent and advanced atherosclerotic vascular diseases than subjects with normal body weight [5]. Weight loss has, therefore, become one of the primary targets in the prevention of CAD. Intentional weight loss has been reported to reduce the risk of adverse clinical outcomes in subjects with CAD.

A number of surgical procedures supported by postoperative diets have recently been developed to facilitate weight loss, particularly for those with morbid obesity. Bariatric surgery and resultant weight loss have been reported to lead to an improvement in endothelial function and coronary microvascular function, thus reducing coronary artery calcification and the prevalence of myocardial infarction in morbidly obese patients[6–8]. Laparoscopic sleeve gastrectomy (LSG) is a novel technique that has been shown to provide sustained weight loss in patients with obesity not responding to dietary measures [9]. Although LSG provides a significant weight loss in morbidly obese patients, its impact on early markers of atherosclerosis has not been studied yet.

Thrombomodulin is a cofactor of thrombin located on the surface of endothelial cells and expressed in response to endothelial cell damage [10]. Thrombomodulin is associated with extensive CAD, stroke, or peripheral arterial disease [11, 12]. Hypercholesterolemia treatment with fluvastatin has been reported to decrease thrombomodulin concentrations [13]. A positive correlation between thrombomodulin concentrations and the body mass index has been reported in obese women [14]. Weight loss following bariatric surgery with Roux-en-Y gastric bypass surgery has been shown to reduce the thrombomodulin concentrations. However, data concerning the role of LSG on thrombomodulin concentrations in morbidly obese subjects is lacking.

This study aimed to investigate the impact of LSG on thrombomodulin concentration and early markers of atherosclerosis.

## 2. Methods

### 2.1. Patient Selection

This prospective study was conducted on patients undergoing LSG in our institute between June 2021 and December 2021. A written informed consent was obtained from all participants before enrollment. The study was approved by the Institutional Review Board and was conducted under the Helsinki declaration. Indications for bariatric surgery were as follows: body mass index (BMI) > 40 kg/m^2^ or BMI > 35 kg/m^2^ in the presence of comorbidities (diabetes, cardio-respiratory disease, severe joint disorders, psychological problems resulting from obesity) that are expected to improve following surgically induced weight loss [15]. LSG was performed in case where nonsurgical approaches including diet, exercise programs and pharmacological therapy failed to induce weight loss. When the desired results cannot be obtained with the mentioned approaches, LSG becomes a treatment option. The LSG procedure is one of the bariatric surgical procedures that reduces approximately 75–80% of the stomach size.

All patients 18 years and older who met the eligibility criteria for bariatric surgery were enrolled in the study. The exclusion criteria were as follows: preexisting CAD, the presence of uncontrolled overt diabetes (HbA1c >7% within the last two months), moderate to severe kidney or liver disease, being under hypercholesterolemia treatment, the presence of moderate to severe valvular dysfunction, left ventricular ejection fraction <50%, presence of an intracardiac device (e.g., pacemaker, ICD), severe mental disorders, and binge eating disorder.

Systolic and diastolic blood pressures were recorded in each patient. Fasting blood samples were collected for blood glucose, lipids, and thrombomodulin measurement before and six months after LSG.

### 2.2. The Carotid Artery Intima-Media Thickness

Carotid artery intima-media thickness (CIMT) measurement was carried out prior to and six months after LSG by the same sonographer with a high-frequency (7.0–13.0 MHz) linear ultrasound scanning probe (Siemens Healthineers, Erlangen, Germany). The left and right common carotid arteries were imaged proximal to the bulb. Manual tracing was used to obtain the mean IMT. Average IMT measurements from three consecutive cardiac cycles were recorded.

### 2.3. Ankle-Brachial Index

The Doppler method of continuous waves was used for the determination of the ABI. The systolic arterial pressure was measured in the dorsalis pedis, the posterior tibial artery, and the brachial artery. The ratio of the systolic pressure at the ankle over the systolic pressure at the arm was calculated.

### 2.4. Primary Outcome

The change in thrombomodulin concentration from baseline (preoperative) to the sixth postoperative month following the LSG was the primary outcome of this study. The relationship between the change in thrombomodulin concentration and BMI, CIMT, ABI, and blood lipids was the study's secondary outcome.

### 2.5. Statistical Analysis

SPSS v21 (SPSS Inc, Chicago, IL, USA) was used for statistical analysis. Data distribution was examined with the Shapiro–Wilk test. Data were presented as the mean ± standard deviation and frequency (percentage) for categorical variables. The change in the variables from baseline to the postoperative six months was compared using the paired samples *t*-test. Correlation analysis was carried out to assess the relationship between the change in thrombomodulin concentration and BMI, CIMT, and blood lipids. A *p* value of <0.05 was accepted as statistically significant. The *r* values between 0.3 and 0.5 were considered weak correlations, *r* values between 0.5 and 0.7 were considered moderate correlations, and *r* values above 0.7 were considered strong correlations.

## 3. Results

**Figure 1:**
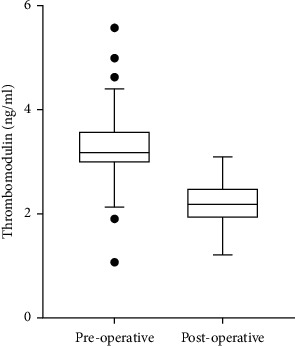
Boxplot graph of preoperative and postoperative thrombomodulin values.

The medical records were available for 44 patients (mean age: 37.2 ± 10.9 years, 65.9% male). Demographic characteristics of the study population are presented in Table 1. Preoperative mean total body weight and BMI were 129 ± 24 kg and 47.1 ± 5.8 kg/m^2^, respectively. LSG led to a significant reduction in total body weight (102 ± 21 kg vs. 129 ± 24 kg, *p* < 0.001) and BMI (37.0 ± 5.6 kg/m^2^ vs. 47.1 ± 5.8 kg/m^2^, *p* < 0.001) at postoperative 6 months when compared with baseline values. No significant changes occurred in fasting plasma glucose, mean platelet volume, neutrophil to lymphocyte ratio, and left ventricular ejection fraction. However, a significant reduction in blood lipids and blood pressure was observed together with the body weight and BMI reduction. There was a significant reduction in thrombomodulin concentration in the postoperative six months compared to the preoperative values (Figure 1). Markers of early atherosclerotic processes, including CIMT and ABI, also improved significantly at the postoperative sixth-month (Table 2).

**Figure 2:**
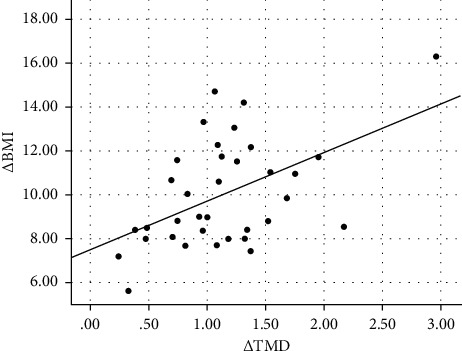
Scatter plot graph of Δ BMI and Δ thrombomodulin values.

**Figure 3:**
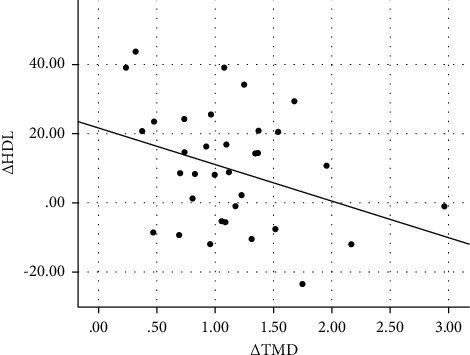
Scatter plot graph of Δ HDL and Δ thrombomodulin values.

**Figure 4:**
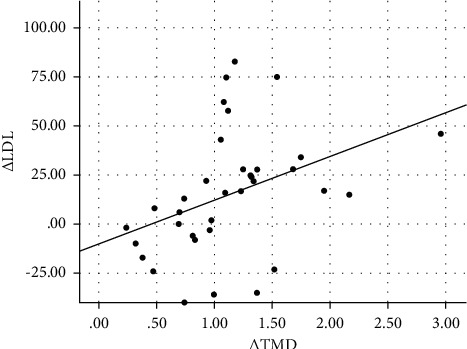
Scatter plot graph of Δ LDL and Δ thrombomodulin values.

**Figure 5:**
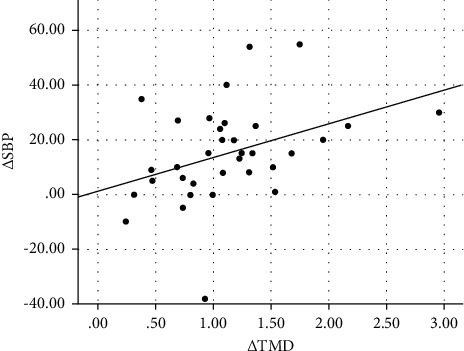
Scatter plot graph of Δ SBP and Δ thrombomodulin values.

**Figure 6:**
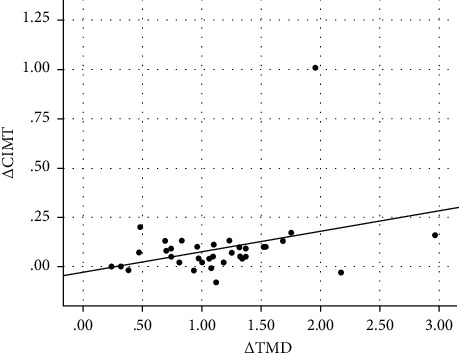
Scatter plot graph of Δ CIMT and Δ thrombomodulin values.

**Figure 7:**
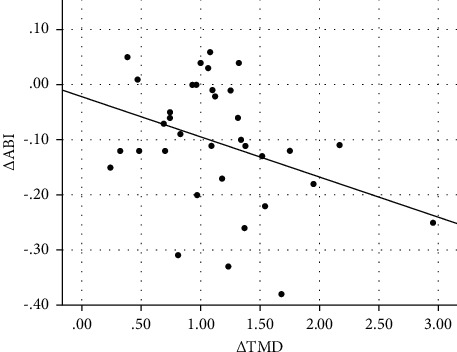
Scatter plot graph of Δ ABI and Δ thrombomodulin values.

Table 3 shows the correlation and selected clinical and laboratory changes of the subjects from baseline to 6 months postoperatively. The change in thrombomodulin concentration (Δ TMD) was significantly correlated with the change in Δ BMI (*r* = 0.500, *p*=0.011), Δ LDL (*r* = 0.389, *p*=0.012), Δ systolic blood pressure (*r* = 0.384, *p*=0.032), and Δ CIMT (*r* = 0.327, *p*=0.022) and was negatively correlated with Δ HDL (*r* = −0.344, *p*=0.020) and Δ ABI (*r* = −0.357, *p*=0.020). Scatter plot graph of Δ thrombomodulin and other parameters are given in Figures 2–7.

## 4. Discussion

The present study demonstrates that LSG leads to significant improvements in blood lipids in addition to systolic and diastolic blood pressures and surrogates of atherosclerotic burden and endothelial function, ABI, and CIMT at postoperative six months. Also, a significant decrease in thrombomodulin levels was observed. Moreover, the reduction in thrombomodulin concentration is significantly correlated with the reduction in BMI, CIMT, and LDL cholesterol levels and increased ABI and HDL. Markers of early atherosclerotic cardiovascular and vascular diseases, including CIMT and ABI, also improved significantly after LSG, which leads us to consider that atherosclerosis is preventable.

Obesity causes a major burden on the healthcare system as an independent risk factor for atherosclerotic cardiovascular disease and its association with several disorders such as type 2 diabetes and hypertension. The loss of excess weight has been shown to decrease the prevalence of atherosclerotic events [16]. There are several treatment options targeting weight loss, including surgical and nonsurgical strategies; however, bariatric surgery delivers the greatest sustainable weight loss. Laparoscopic sleeve gastrectomy has emerged as a technique in the surgical management of subjects with excess body weight as a stand-alone procedure due to its feasibility and safety [17–19]. LSG has been shown to provide an excess weight loss of up to 82.9% [20]. A recent meta-analysis with 796 obese individuals has revealed that subjects undergoing bariatric surgery lose an additional 26 kg compared to the subjects receiving nonsurgical treatment [21]. Moreover, the weight loss achieved with bariatric surgery may be preserved for up to 20 years [22]. Bariatric surgery has also been reported to modify the risk factors associated with CAD through a reduction in the prevalence of hypertension and dyslipidemia [23–25].

Proinflammatory state existing in subjects with obesity and blood concentrations of the inflammatory markers, including C-reactive protein, interleukin-6, adipokines, fibrinogen, and PAI-1, are shown to be reduced following bariatric surgery. In a cardiovascular event, a tissue-type plasminogen activator (T-PA), which causes the onset of intravascular fibrinolysis, is acutely secreted from endothelial cells. Thus, thrombus formation occurs. Fibrin degradation is achieved by TPA activation that transforms plasminogen to plasmin. PAI-1 mainly inhibits t-PA. It has been reported in various studies that the effectiveness of PAI-1 levels may be decreased by reducing fat mass via surgical resection or a diet [26].

The effects of inflammation on endothelial damage at the early stage of the atherosclerosis process are well-known. Therefore, all inflammatory mechanisms have adverse effects on endothelial function [27].

Thrombomodulin is a 557 amino acid residue, type 1 transmembrane glycoprotein, localized primarily to the vascular endothelium [28]. Thrombomodulin has been shown to modulate coagulation, innate immunity, inflammation, and cell proliferation. It works as a ligand for thrombin and is a critical cofactor for the major natural anticoagulant protein C (PC) system [29]. Recent evidence indicates that thrombomodulin has additional properties beyond its anticoagulant effects. Thrombomodulin delivers cytoprotective effects on the vascular endothelium and is released in response to stress-induced endothelial damage [30]. Thus, high levels of thrombomodulin can be used as a biomarker for endothelial injury, which is the early phase of atherosclerosis. A recent study on 60 patients undergoing Roux-en-Y gastric bypass surgery has shown that a significant reduction in soluble thrombomodulin occurs one year after bariatric surgery [31]. In another study evaluating 66 patients who underwent Roux-en-Y gastric bypass surgery, it was shown that it could prevent atherogenesis in the early stages by breaking the vicious circle between inflammation and endothelial dysfunction [32].

A marked improvement in endothelial function and low-grade inflammation was observed in morbidly obese patients who lost weight after bariatric surgery [33]. However, data concerning the change in thrombomodulin concentration after LSG and its association with endothelial dysfunction in subjects undergoing LSG has not been studied yet.

To the best of our knowledge, this study is the first to demonstrate the reduction in thrombomodulin and the improvement in ABI and CIMT 6 months after LSG. The results of this study suggest that weight loss achieved with LSG leads to a significant improvement in early markers of atherosclerosis, as indicated by the reduction in thrombomodulin and CIMT and the increase in ABI. Our findings also show that the reduction in thrombomodulin concentration following the LSG is correlated with improvements in blood lipids and systolic blood pressure. Given that thrombomodulin is released as a response to endothelial damage, we speculate that LSG leads to a significant improvement in preexisting endothelial dysfunction in morbidly obese subjects. As a result of the improvement caused by LSG, atherosclerosis can be considered to be regressed or stopped, and thrombomodulin may be used as a tracking marker in the following years. However, further research with a larger sample size is required to address the role of thrombomodulin in subjects undergoing LSG. Therefore, we do not recommend the routine use of thrombomodulin concentration after bariatric surgery for now.

The relatively small sample size of this study is one of the limitations to be mentioned. However, the strict indications recommended by the international guidelines restrict the implementation of LSG only to subjects who failed to lose weight with nonsurgical approaches. Therefore, the published series for bariatric surgery includes a limited number of patients. Second, we focused on the endothelial role of thrombomodulin. However, it also plays a role in inflammation and the coagulation cascade. The lack of data regarding the change in inflammatory markers and coagulation tests is also a limitation for our study. We believe that the correlations between markers other than BMI are rather weak; however, we chose to report these correlations since the relationship between delta values of BMI and thrombomodulin indicates a somewhat reliable relationship. Finally, the weak-to-moderate correlations demonstrated in the study are another limitation, and therefore, the results and demonstrated relationships should be evaluated cautiously.

## 5. Conclusion

LSG leads to significant improvements in blood lipids, systolic and diastolic blood pressure, and surrogate markers of atherosclerotic burden and endothelial function, including thrombomodulin, ABI, and CIMT, at postoperative six months. LSG might prevent or reduce atherogenesis in the early stages by stopping endothelial dysfunction.

## Figures and Tables

**Table 1:** Demographic features, baseline laboratory measurements, and clinical characteristics of the study population.

	*n* = 44
Age, years	37.2 ± 10.9
Gender, male	29 (65.9%)
Weight, kg	129 ± 24
Body mass index, kg/m^2^	47.1 ± 5.8
Hypertension, *n*	16 (36.4%)
Diabetes, *n*	15 (34.1%)
Smoking, *n*	11 (25.0%)
Leukocyte count, 10^3^/mm^3^	8.4 ± 1.9
Fasting glucose, mg/dl	102 ± 17
Creatinine, mg/dl	0.66 ± 0.13
Total cholesterol, mg/dl	193 ± 30
HDL cholesterol, mg/dl	35.1 ± 11.2
Triglyceride, mg/dl	247 ± 69
LDL cholesterol, mg/dl	107 ± 28
EF, %	62.6 ± 2.9
Systolic blood pressure, mmHg	136 ± 13
Diastolic blood pressure, mmHg	80 ± 9
Heart rate, beats/min	78 ± 15

Data are presented as the mean ± standard deviation for continuous variables and as frequency (%) for categorical variables. EF = Ejection fraction; HDL = High-density lipoprotein; LDL = Low-density lipoprotein; TG = Trigylceride.

**Table 2:** The comparison of the clinical, laboratory, and echocardiographic parameters from baseline to the postoperative 6 months.

	Baseline	Postoperative 6 months	Change (Δ)	*p* value
Weight, kg	129 ± 24	102 ± 21	27 ± 7	**<0.001**
Body mass index, kg/m^2^	47.1 ± 5.8	37.0 ± 5.6	10.1 ± 2.4	**<0.001**
Fasting plasma glucose, mg/dl	102 ± 17	99 ± 16	3.3 ± 36	0.102
Mean platelet volume, fL	7.5 ± 1.6	7.8 ± 1.3	−0.3 ± 1.67	0.120
NLR	1.6 ± 0.8	1.8 ± 0.8	−0.2 ± 1	0.172
HDL cholesterol, mg/dL	35.1 ± 11.2	41.5 ± 10.7	−6.3 ± 19.4	**0.006**
Triglyceride, mg/dL	247 ± 69	176 ± 67	70 ± 112	**<0.001**
LDL cholesterol, mg/dL	109 ± 27	89 ± 16	19 ± 34	**0.001**
EF, %	62.4 ± 2.4	62.5 ± 2.4	−0.1 ± 1	0.534
SBP, mmHg	136 ± 13	123 ± 15	13 ± 20	**<0.001**
DBP, mmHg	80 ± 9	75 ± 8	5 ± 11	**0.022**
CIMT, mm	1.06 ± 0.05	0.98 ± 0.14	0.1 ± 0.2	**0.003**
ABI	0.85 ± 0.08	0.95 ± 0.05	−0.1 ± 0.1	**<0.001**
Thrombomodulin, ng/mL	3.3 ± 0.8	2.2 ± 0.4	1.1 ± 0.7	**<0.001**

Data are presented as the mean ± standard deviation. ABI = Ankle-brachial index; CIMT = Carotid intima-media thickness; DBP = Diastolic blood pressure; EF = Ejection fraction; HDL = High-density lipoprotein; LDL = Low-density lipoprotein; NLR = Neutrophil to lymphocyte ratio; SBP = Systolic blood pressure; TG = Trigylceride.

**Table 3:** Correlation analysis between Δ thrombomodulin, and Δ carotid intima-media thickness, Δ ankle-brachial index, and several clinical and laboratory variables.

	Δ Thrombomodulin	
	*r*	*p*
Δ BMI	0.500	**0.001**
Δ HDL	−0.344	**0.022**
Δ Triglyceride	−0.065	0.681
Δ LDL	0.389	**0.011**
Δ SBP	0.384	**0.012**
Δ DBP	0.026	0.473
Δ CIMT	0.327	**0.032**
Δ ABI	−0.357	**0.020**

Delta (Δ) values were obtained by subtracting sixth-month values from the baseline values. ABI = Ankle-brachial index; BMI = Body mass index; CIMT = Carotid intima-media thickness; DBP = Diastolic blood pressure: HDL = High-density lipoprotein; LDL = Low-density lipoprotein; SBP = Systolic blood pressure; TG = Triglyceride, *p* < 0.05 and *r* > 0.7 was accepted significant.

## Data Availability

No data were used to support this study.
